# Minor Isozymes Tailor Yeast Metabolism to Carbon Availability

**DOI:** 10.1128/mSystems.00170-18

**Published:** 2019-02-26

**Authors:** Patrick H. Bradley, Patrick A. Gibney, David Botstein, Olga G. Troyanskaya, Joshua D. Rabinowitz

**Affiliations:** a Department of Molecular Biology, Princeton University, Princeton, New Jersey, USA; b Department of Computer Science, Princeton University, Princeton, New Jersey, USA; c Department of Chemistry, Princeton University, Princeton, New Jersey, USA; d Lewis-Sigler Institute for Integrative Genomics, Princeton University, Princeton, New Jersey, USA; Pacific Northwest National Laboratory

**Keywords:** cerevisiae, duplicates, genomics, isozymes, metabolomics, paralogs, systems biology, transcriptomics, yeast

## Abstract

Isozymes are enzymes that differ in sequence but catalyze the same chemical reactions. Despite their apparent redundancy, isozymes are often retained over evolutionary time, suggesting that they contribute to fitness. We developed an unsupervised computational method for identifying environmental conditions under which isozymes are likely to make fitness contributions. This method analyzes published gene expression data to find specific experimental perturbations that induce differential isozyme expression. In yeast, we found that isozymes are strongly enriched in the pathways of central carbon metabolism and that many isozyme pairs show anticorrelated expression during the respirofermentative shift. Building on these observations, we assigned function to two minor central carbon isozymes, aconitase 2 (*ACO2*) and pyruvate kinase 2 (*PYK2*). *ACO2* is expressed during fermentation and proves advantageous when glucose is limiting. *PYK2* is expressed during respiration and proves advantageous for growth on three-carbon substrates. *PYK2*’s deletion can be rescued by expressing the major pyruvate kinase only if that enzyme carries mutations mirroring *PYK2*’s allosteric regulation. Thus, central carbon isozymes help to optimize allosteric metabolic regulation under a broad range of potential nutrient conditions while requiring only a small number of transcriptional states.

**IMPORTANCE** Gene duplication is one of the main evolutionary paths to new protein function. Typically, duplicated genes either accumulate mutations and degrade into pseudogenes or are retained and diverge in function. Some duplicated genes, however, show long-term persistence without apparently acquiring new function. An important class of isozymes consists of those that catalyze the same reaction in the same compartment, where knockout of one isozyme causes no known functional defect. Here we present an approach to assigning specific functional roles to seemingly redundant isozymes. First, gene expression data are analyzed computationally to identify conditions under which isozyme expression diverges. Then, knockouts are compared under those conditions. This approach revealed that the expression of many yeast isozymes diverges in response to carbon availability and that carbon source manipulations can induce fitness phenotypes for seemingly redundant isozymes. A driver of these fitness phenotypes is differential allosteric enzyme regulation, indicating isozyme divergence to achieve more-optimal control of metabolism.

## Abstract (precis)

Gene duplication is one of the main evolutionary paths to new protein function. Typically, duplicated genes either accumulate mutations and degrade into pseudogenes or are retained and diverge in function. Some duplicated genes, however, show long-term persistence without apparently acquiring new function. An important class of isozymes consists of those that catalyze the same reaction in the same compartment, where knockout of one isozyme causes no known functional defect. Here we present an approach to assigning specific functional roles to seemingly redundant isozymes. First, gene expression data are analyzed computationally to identify conditions under which isozyme expression diverges. Then, knockouts are compared under those conditions. This approach revealed that the expression of many yeast isozymes diverges in response to carbon availability and that carbon source manipulations can induce fitness phenotypes for seemingly redundant isozymes. A driver of these fitness phenotypes is differential allosteric enzyme regulation, indicating isozyme divergence to achieve more-optimal control of metabolism.

## INTRODUCTION

Isozymes are distinct proteins within a single organism that can catalyze the same biochemical reactions. Although some isozymes differ in localization, substrate specificity, or cofactor preference, there are also many isozymes that are not differentiated by these criteria. The genome of budding yeast (Saccharomyces cerevisiae) contains many duplicate genes encoding isozymes that have persisted since the ancient duplication of the whole genome that led to the evolution of the modern *Saccharomyces* (1). Only a small fraction of these yeast gene duplications remain, strongly suggesting that the remaining ones, including those that encode isozymes, must somehow have contributed to evolutionary fitness.

Several explanations, both complementary and at times conflicting, have been advanced for the retention of such isozymes (and of gene duplicates more generally). One is gene dosage, in which multiple gene copies contribute to maintaining adequate total enzyme levels. Papp et al. have argued that many isozyme pairs can be explained by gene dosage, since in a flux-balance model reactions catalyzed by isozymes tended to carry higher flux (2). However, a subsequent study using experimentally determined fluxes estimated that less than 20% of isozyme pairs catalyzed high-flux reactions (3). Additionally, in some high-flux reactions, such as aconitase and pyruvate kinase reactions, one “major” isozyme but not the other “minor” isozyme has been found to be essential under laboratory conditions.

Another potential explanation involves genetic backup, i.e., the ability of isozymes to compensate for the deletion of their partners. However, since genetic backup cannot be directly selected, it is generally agreed that this is more likely to be a side effect of isozyme retention than the cause (4). Kafri et al. demonstrated that some isozymes change in expression after deletion of their partners (“transcriptional reprogramming”) and argued that selection for robustness against nongenetic noise could give rise to both transcriptional reprogramming and genetic backup (5, 6); however, a follow-up study reported that transcriptional reprogramming was confirmed in only ∼11% of tested isozyme pairs (7).

Isozymes are often differentially regulated, suggesting a role in fine-tuning metabolic capabilities (8). A well-understood example of such fine-tuning involves the seven hexose transporters of S. cerevisiae (*HXT1* to *HXT7*), some of which are high-affinity/low-flux transporters and others low-affinity/high-flux transporters. Collectively, these transporters allow yeast to import hexose optimally under a wide variety of environmental conditions (9). Another form of fine-tuning involves optimization for growth under specific (and less commonly studied) environmental conditions, and it has been argued that isozymes contribute to such optimization (2, 10, 11). However, thus far, existing computational and experimental tools have not proven to be well suited to finding the most relevant environmental conditions for explaining the existence of isozymes. For example, flux balance analysis (FBA) models metabolism at the level of reactions, not genes, and is therefore intrinsically unable to differentiate between isozymes (2, 12, 13).

High-throughput fitness assays are, in principle, well suited to identifying the function of isozymes. Most isozymes have been knocked out in Saccharomyces cerevisiae and the growth rate and competitive fitness of the resulting strains measured (3, 11, 14–16). A large number of isozyme deletions, however, have failed to show substantial fitness defects under laboratory growth conditions. For example, in a recent study that measured competitive fitness with high precision, 65% of the assayed isozyme deletions had relative fitness levels of ≥0.99 and some “minor” isoforms, such as the pyruvate kinase isozyme *PYK2*, even showed a slight fitness advantage (15). A limitation of such studies, including those that go beyond single gene deletions to measure fitness defects of double deletion strains (17) or triple deletion strains (18), is that they have been conducted under only a few environmental conditions, mainly using growth on rich media or defined media with amino acids, with glucose (and sometimes ethanol [EtOH] or glycerol) as the carbon source. The genetic tools that enable these massively parallel assays also tend to use amino acid auxotrophies as selectable markers; growth of these knockout strains therefore requires nutritional supplements that can themselves contribute to growth, which is less than ideal for studying the function of genes in central metabolism (19).

In contrast, transcriptional profiling has been conducted under a much wider array of experimental conditions. Thus, an alternative approach to identifying the function of isozymes is to mine compendia of gene expression data, with the aim of identifying conditions under which isozymes may contribute to fitness. Indeed, previous studies have noted that the differential expression of isozymes is a feature of many microarray experiments (20, 21). However, existing expression analyses (5, 21) have tended to focus on identifying transcriptional coregulation of isozymes with other enzymes or processes. They have not focused on generating hypotheses about which environments are specifically associated with isozyme expression divergences (and hence potentially with function).

Here we developed methods for systematically associating isozymes with specific environmental perturbations and used these methods to identify an important role for isozymes in adapting to changes in carbon source availability. This observation is intriguing given that many (though not all) metabolic isozymes date from the putative events that might have led *Saccharomyces* to adopt a bifurcated lifestyle, primarily fermenting when glucose is present and respiring otherwise (in what is called the “Crabtree effect”) (22–24). It suggests a rationale for the retention of isozymes over evolutionary time, providing flexibility to central metabolism and, in particular, to central carbon metabolism. We tested for such flexibility experimentally, by growing cells lacking specific isozymes in alternative carbon sources. In two cases, we found growth defects for isozyme deletions on nonstandard carbon sources, associating for the first time a specific functional role with the gene(s) that encode the function. These experimental results provide support for the idea that central carbon metabolic isozymes have been retained over evolutionary time to optimize the metabolism of diverse carbon sources.

## RESULTS

### Differentially expressed isozymes are prevalent in central carbon metabolism.

We began by assembling a list of metabolic isozymes (see Materials and Methods). Among 77 metabolic isozyme pairs in yeast, we found that 24 were differentiated by compartment, e.g., mitochondria versus cytosol, leaving 53 colocalized metabolic isozymes. We found that, like duplicated yeast genes in general (24), these 53 isozymes are enriched for genes involved in central carbon metabolism (see Fig. S1 in the supplemental material) (Fisher’s test *P = *5.7 × 10^−7^). Nearly every step in glycolysis and gluconeogenesis can be catalyzed by two or more enzymes, and storage carbohydrate metabolism and the pentose phosphate pathway also contain many isozyme pairs. In contrast, while many metabolic enzymes are involved in amino acid *de novo* biosynthesis, these pathways contain comparatively few isozymes. Indeed, in the Yeast Pathway database (25), 8% (51/632) of reactions overall are shown to be catalyzed by isozymes; however, in the pathways of glycolysis, gluconeogenesis, and fermentation, this number rises to 67% (8/12; Bonferroni-Holm-corrected Fisher’s test *P* = 5.6 × 10^−4^). The pentose phosphate pathway and tricarboxylic acid (TCA)/glyoxylate cycle are also enriched for reactions catalyzed by isozymes, but these were not significant after correction (Bonferroni-Holm corrected Fisher’s test *P* = 0.15 and *P* = 0.092, respectively; see Table S1 in the supplemental material).

10.1128/mSystems.00170-18.2 FIG S1 Colocalized metabolic isozymes in Saccharomyces cerevisiae are enriched in central carbon metabolism. Reactions catalyzed by metabolic isozymes of the same compartment are indicated with black arrows; metabolites that are substrates or products of those isozyme-catalyzed reactions are shown as white circles. Other reactions are indicated with a gray dashed line. Reactions are highlighted according to pathway (inset). While a large proportion of reactions catalyzed by these isozymes correspond to central carbon metabolism (glycolysis and gluconeogenesis, the TCA cycle, and the pentose phosphate pathway), comparatively few correspond to, for example, amino acid biosynthesis (green). Enrichment *P* values are given in Table S1. Download FIG S1, EPS file, 0.5 MB. Copyright © 2019 Bradley et al. 2019 Bradley et al. This content is distributed under the terms of the Creative Commons Attribution 4.0 International license.

10.1128/mSystems.00170-18.7 TABLE S1 Enrichments of metabolic pathways for reactions catalyzed by isozymes. Pathways were drawn from the Yeast Pathway database (25). *P* values are from two-tailed Fisher exact tests (Bonferroni-Holm corrected for multiple testing). Pathway sets that were significantly enriched for isozymes at *P* values of <0.05 are shown in bold. Download Table S1, DOCX file, 0.01 MB. Copyright © 2019 Bradley et al. 2019 Bradley et al. This content is distributed under the terms of the Creative Commons Attribution 4.0 International license.

We also note that metabolic isozymes were strongly enriched for genes dating from the whole-genome duplication (WGD) of yeast (65% of isozymes date from the WGD, compared with 19% of the genome; Fisher’s test *P* < 10^−22^). Compared to non-WGD yeasts, post-WGD yeasts such as Saccharomyces cerevisiae are more likely to exhibit the Crabtree effect, i.e., are more likely to ferment glucose to ethanol even in the presence of oxygen. In addition, post-WGD yeast are more likely to be able to survive without the mitochondrial genome (i.e., to be “petite positive”) (22). These observations raise the possibility that selective pressures related to carbon metabolism, and, in particular, to transitions between fermentation and respiration, may have driven the retention of metabolic isozymes.

We wanted to determine whether isozyme pairs tend to act together as a functional unit or whether, alternatively, each isozyme has a discrete role. If the former is the case, we would expect a strong tendency for isozymes to be coexpressed; if the latter, we would expect anticorrelation or no correlation in expression. To address this issue, we assembled a large compendium of gene expression data consisting of more than 400 data sets (each comprising at least 6 arrays) and calculated the correlation of each isozyme gene pair’s expression within each data set.

We focused on identifying negative correlations between isozymes across arrays from single experimental data sets, in contrast to looking at the expression levels of genes across all arrays in the compendium, for three main reasons. First, gene expression levels are difficult to compare across experiments from different laboratories and different array technologies, and our compendium includes several different single- and double-channel array platforms. In contrast, gene correlations calculated within single experiments can be made comparable using relatively simple normalization procedures (26, 27). Gene correlations within data sets have also been shown to be more informative about function than correlations computed across an entire compendium (26, 27). Second, we expect negative correlation of isozyme expression to be observed during experiments that capture the transition between environments where one isozyme is preferred to another. Typical biclustering methods discard information about which arrays come from a single experiment and therefore can group single arrays from unrelated experiments, a result that is less straightforward to interpret. Third, when gene transcripts are measured by microarray, cross-hybridization can occur for genes with high nucleotide sequence identity (28, 29), leading to artifactual positive correlation. Given that many isozyme pairs in yeast have closely related nucleotide sequences, focusing on negative correlation mitigates this technical bias.

**FIG 1:**
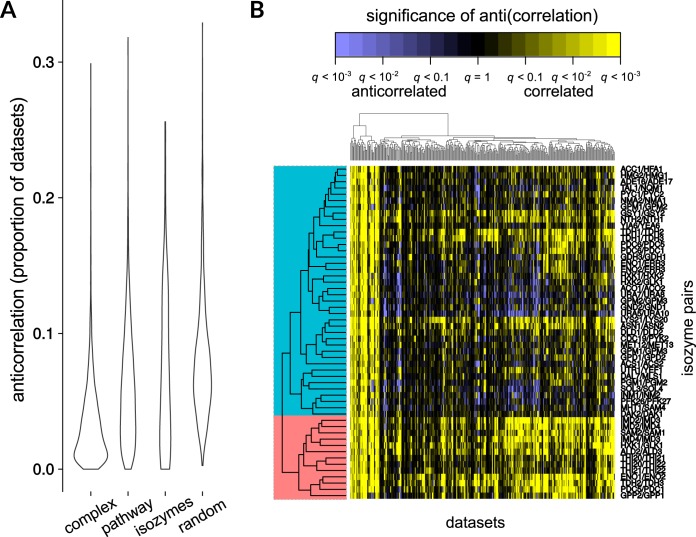
Many isozyme pairs are differentially expressed. (A) Box plots of anticorrelation among isozyme pairs compared with (i) members of the same protein complex, (ii) members of the same metabolic pathway, and (iii) random gene pairs. Isozyme pairs are more likely to show differential expression than genes in the same complex (Bonferroni-Holm-corrected 2-sided Wilcox test *P* value, 1.0 × 10^−4^) but less likely than random genes (Holm-corrected *P* = 0.031). (B) Isozyme pairs separate into two broad categories, depending on how often they are anticorrelated. The matrix displayed shows the correlation (yellow) or anticorrelation (blue) of isozyme pairs (rows) for every data set (columns) in the compendium. Intensity data correspond to the significance of the (anti)correlation (*q* value). Hierarchical clustering performed using an uncentered Pearson’s correlation reveals two main clusters of isozyme pairs; a minority are strongly correlated over most of the compendium, while the majority show condition-dependent anticorrelation.

In our compendium, we found that, overall, isozymes appeared to be anticorrelated less often than random gene pairs (Bonferroni-Holm-corrected Wilcox test *P = *0.031) and more often than members of the same protein complex (*P* = 1.0 × 10^−4^) but did not differ significantly from other genes within the same metabolic pathway (*P* = 0.83; Fig. 1A). When the correlation of isozyme pairs over the entire expression compendium was visualized, it became clear that this intermediate level of anticorrelation could be explained by the existence of two distinct clusters of isozymes; a minority of isozyme pairs appeared to be highly correlated across most of the compendium, while a majority showed strong anticorrelation under a subset of conditions (Fig. 1B). Based on how often (i.e., in how many experiments) an isozyme pair was observed to show anticorrelated expression (false-discovery-rate [*q*] value of ≤0.1), we used logistic regression (see Materials and Methods) to classify the pair as either more like genes from the same protein complex (consistent with a role in dosage) or more like a pair of randomly selected genes (suggesting independent roles for the individual isozymes). We found that 17 isozyme pairs resembled random pairs ≥6× more closely than they resembled pairs drawn from the same protein complexes; at the same threshold, 9 pairs more closely resembled members of the same protein complex (Fig. S2A).

10.1128/mSystems.00170-18.3 FIG S2 (A) Illustration of logistic-regression-based classification of isozyme pairs. This classification relies on the average anticorrelation as defined in Materials and Methods under “Comparison of isozymes with other types of proteins.” The estimated distributions of average anticorrelation are plotted for pairs in the same protein complex (red) and for random pairs (blue), with 95% confidence intervals plotted in light red and light blue, respectively. Blue and red dashed vertical lines represent individual isozyme pairs. Light red isozymes were classified as being more like “complex” pairs [*P*(*C*) > 0.5], with dark red isozymes classifying particularly strongly as “complex” pairs [*P*(*C*) > 0.83]. Light blue and dark blue isozymes represent isozymes that were the same for “random” pairs [*P*(*C*) < 0.5 and *P*(*C*) < 0.17, respectively). Selected isozyme pairs are labeled in red or blue. (B) Partitioning around medoids (PAM) clustering of the binary differential expression matrix described in Materials and Methods (under “Processing of gene expression data”) with three clusters revealed two coherent clusters, shown here. Black cells indicate that a given gene pair (row) was significantly anticorrelated (false-discovery rate [*q*] < 0.1) in a particular data set (column). Within each cluster, columns are sorted from highest to lowest level of anticorrelation of isozyme pairs. The columns with the highest level of anticorrelation of isozyme pairs for each cluster are highlighted on the right-hand side of the figure. These data sets support the idea of a role for these isozyme pairs in the response to the availability of glucose versus other carbon sources. Download FIG S2, EPS file, 2.2 MB. Copyright © 2019 Bradley et al. 2019 Bradley et al. This content is distributed under the terms of the Creative Commons Attribution 4.0 International license.

As described in the introduction, one explanation for isozyme retention reflects gene dosage; that is, having multiple copies of an enzyme may enable increased total enzyme expression (30). If isozymes were retained strictly for the purpose of increased dosage, we would not expect them to be differentially expressed. The prominence of anticorrelated pairs therefore demonstrates that gene dosage alone does not explain the continued retention of the majority of our 53 isozyme pairs, in contrast to some previous assertions (2) but in accord with those by Ihmels et al. (10). Further, duplicated genes can evolve at either similar or divergent rates; we would expect this asymmetry to be higher for functionally divergent genes and less pronounced for the pairs best explained by dosage (31–33). Indeed, when we estimated the levels of this asymmetry, we found that genes in the more “complex-like” isozyme pairs that we identified had evolved at more similar rates than “random-like” pairs (Wilcox test *P = *0.0053), a result that is in line with previous literature comparing divergence in sequence to divergence in function (31–33). Additionally, there was little overlap of coexpressed metabolic isozymes and those isozymes catalyzing high-flux reactions, as defined in a previous study (3), further arguing against a predominant role for dosage for this set of genes; only the GAPDH (glyceraldehyde-3-phosphate dehydrogenase; *TDH1-3*) and hexokinase (*HXK1*/*GLK1*) enzymes appeared in both lists. Indeed, it appears that a majority of isozyme pairs are strongly anticorrelated in a condition-dependent manner, suggesting a role for these pairs in adaptation to different environments.

### A set of 21 isozyme pairs shows strong differential expression with changing carbon availability.

Visualizing the anticorrelation of isozyme pairs also revealed that many were differentially expressed in the same data sets. This suggested that specific experimental conditions may be particularly relevant to explaining isozyme retention. We therefore wanted to identify the specific experimental perturbations leading to isozyme expression anticorrelation. Building on related work (5, 21, 34) (see also the note in Text S1 in the supplemental material), we first simply sorted the transcriptional data sets (each containing several individual arrays; for example, a heat shock time course would represent one “data set” [27]) by the number of isozyme pairs in each that were anticorrelated. Data sets with the highest levels of differential expression of isozyme pairs included those from many experiments related to the carbon source (Table S2). An alternative analysis by partitioning around medoids (PAM) clustering of the differential expression matrix revealed similar results (Fig. S2B).

10.1128/mSystems.00170-18.1 TEXT S1 Supplemental methods and key differences from previous analyses of expression data. Download Text S1, DOCX file, 0.03 MB. Copyright © 2019 Bradley et al. 2019 Bradley et al. This content is distributed under the terms of the Creative Commons Attribution 4.0 International license.

10.1128/mSystems.00170-18.8 TABLE S2 Ten data sets with the greatest number of anticorrelated isozyme pairs. The source of the data set (NP, not published), a brief description of the data set, and a list of the anticorrelated isozymes are provided. The majority of these data sets have clear connections to glucose signaling (Ras, protein kinase A [PKA]) and availability. (Note that the *sua*5Δ deletion lacks cytochrome *c* and cannot grow on respiratory media.) Download Table S2, DOCX file, 0.01 MB. Copyright © 2019 Bradley et al. 2019 Bradley et al. This content is distributed under the terms of the Creative Commons Attribution 4.0 International license.

If anticorrelation between isozymes were really associated with a specific experimental perturbation, then experiments involving similar perturbations should be more likely to show isozyme anticorrelation, while unrelated experiments should be less likely. To identify cases where both criteria were true, we first needed to assess the relatedness of the experiments in our compendium. We reasoned that similar experimental perturbations should cause similar genes to be differentially expressed. Therefore, we grouped the experiments into clusters, based on which genes showed the strongest expression changes. This was accomplished by quantifying the variance of each gene within each data set and then clustering the variance vectors using consensus k-means clustering, with the number of clusters determined by Akaike information criterion (AIC) analysis (35) (see Materials and Methods). This method was effective at grouping data sets reflecting similar experimental perturbations. For example, one cluster of data sets included diauxic shift time courses (36–38), carbon starvation time courses (39), a panel of mutants with and without glucose (40), and a 15-day wine fermentation (41).

**FIG 2:**
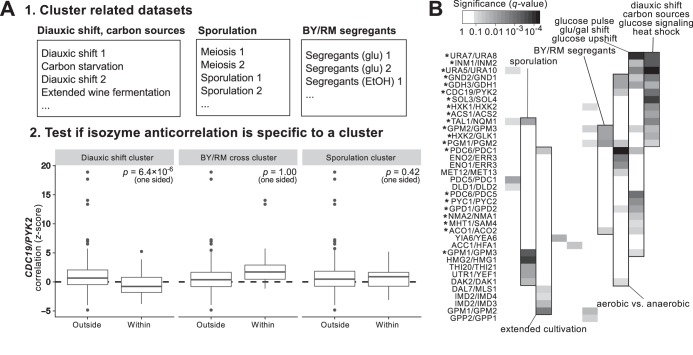
Twenty-one isozyme pairs are associated with the metabolism of alternative carbon sources. (A) Outline of method for association of isozyme pairs with particular data set clusters. In step 1, data sets are grouped into clusters of related experimental conditions (see Materials and Methods). Three of the resulting clusters are shown, with representative data sets. In step 2, for each data set cluster and each isozyme pair, we test whether that pair is anticorrelated within that cluster, and if so, whether it is significantly more anticorrelated within that cluster than in other data sets. We show as an example the *CDC19*/*PYK2* pair, which passes these criteria only within the first cluster of data sets (related to diauxic shift). This suggests that the *CDC19*/*PYK2* pair is associated with the respirofermentative transition. BY/RM, a cross of the lab strain BY4716 and the wine strain RM-11 (95); glu, glucose. (B) A set of 21 isozyme pairs was specifically differentially expressed in two clusters of data sets having to do with metabolism of alternative carbon sources. Separately, 5 other pairs appeared to be associated with sporulation and meiosis. Each filled cell indicates that significant anticorrelation of the given isozyme pair was observed within a given data set cluster, with the intensity of the cell corresponding to the *q* value (the false-discovery-rate analog of a *P* value). Asterisks denote the 21 isozyme pairs significantly associated with the diauxic shift and glucose perturbations. gal, galactose.

After clustering these data sets, we could identify clusters of similar experiments in which the correlation of isozyme pairs was consistently negative. Additionally, to ensure that the associations that we found were not only associated with but also specific to an experimental condition, we also required that isozyme pairs be significantly more differentially expressed in a given cluster of experiments than in experiments from the rest of the compendium, using a one-tailed rank sum test (see Fig. 2A and Materials and Methods).

Indeed, we found that a core set of 13 isozyme pairs tended to be particularly strongly anticorrelated under a cluster of conditions having to do with diauxic shift/glucose limitation, and a partially overlapping set of 14 pairs was strongly anticorrelated in a cluster of data sets containing several glucose pulse/upshift experiments; those pairs numbered 21 in total (Fig. 2B). We also found that, for instance, 6 isozyme pairs were specifically associated with meiosis and sporulation and 9 pairs with aerobic versus anaerobic growth. In all, these analyses suggest that 21 of our 53 pairs were differentiated primarily by gene expression on carbon sources and 16 by gene expression under other conditions, leaving 16 pairs that were not associated with a cluster of experiments. These findings highlight the ability of this method, applied to a large expression compendium, to associate subgroups of anticorrelated isozymes not only with stress in general but also with more-specific environmental stressors.

**FIG 3:**
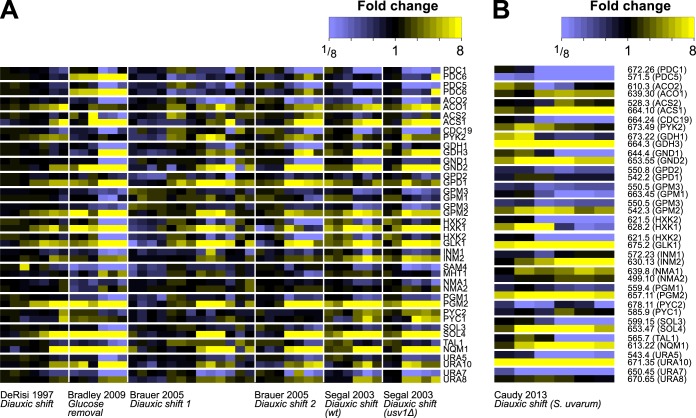
Anticorrelation of 21 isozyme pairs in response to glucose availability. (A) Gene expression profiles of isozymes that are associated with the transition from using glucose to using alternative carbon sources. Array data were collected from several diauxic shift and carbon removal experiments; data show induction of one member of the pair (yellow) and repression of the other (blue) across the diauxic shift. Intensity data correspond to fold change. Genes are grouped into isozyme pairs. (B) Gene expression signatures of isozymes are conserved over evolutionary time. Isozymes were mapped to their syntenic orthologs in Saccharomyces uvarum. The data representing expression of these orthologs in S. uvarum during the diauxic shift (94) show the same overall pattern as that seen with the original isozymes in Saccharomyces cerevisiae (compare panel A).

Examining the original expression data from diauxic shift and glucose removal experiments revealed a clear visual pattern of anticorrelation (Fig. 3A) which was conserved across different yeast strains (Fig. S3) (42) and even across species, as shown using data from the most diverged *Saccharomyces sensu stricto* yeast, Saccharomyces bayanus (now called Saccharomyces uvarum) (Fig. 3B) (43). Taken together, these results suggest that a core set of central carbon metabolic isozymes may be involved in adaptation to nonfermentable carbon sources.

**FIG 4:**
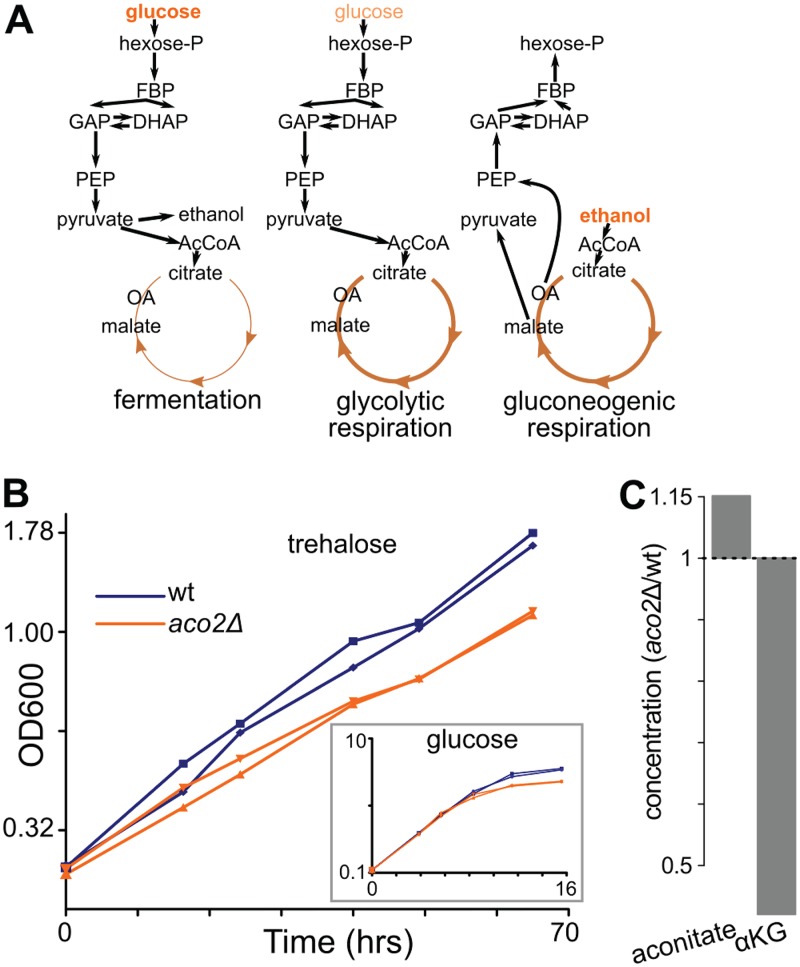
Deletion of the minor aconitase isozyme *aco2* results in a selective growth defect on trehalose, indicating impaired glycolytic respiration. (A) Schematic of metabolism across the diauxic shift. In the presence of high levels of glucose (left), S. cerevisiae prefers to ferment glucose to ethanol. As glucose becomes limiting (center), S. cerevisiae continues to use glucose but converts it into acetyl-CoA (AcCoA) and, eventually, CO_2_, in so doing driving TCA cycle turning and oxidative phosphorylation. We term this state “glycolytic respiration.” Finally, when glucose is exhausted, S. cerevisiae uses ethanol to make acetyl-CoA as well as sugar phosphates through gluconeogenesis. We refer to this state as gluconeogenic respiration. DHAP, dihydroxyacetone phosphate; GAP, glyceraldehyde-3-phosphate; FBP, fructose-1,6-bisphosphate; PEP, phosphoenolpyruvate; OA, oxaloacetate. (B) Growth of wild-type (wt) and *aco2Δ* strains on minimal medium with trehalose, which is digested extracellularly to provide a steady but limiting amount of glucose, revealed a growth defect for the *aco2Δ* mutant during gluconeogenic respiration. In contrast, under conditions of growth on glucose (inset), the *aco2* deletion mutant had no growth defect in the log phase and began to show a growth defect only when glucose became limiting. Data represent biological duplicates. (C) During steady-state growth under conditions of limiting glucose, the level of aconitate was slightly elevated (115% of the wild-type level) and that of α-ketoglutarate decreased (45% of the wild-type level) in the *aco2Δ* mutant compared to the wild type. Bar plots represent averages of results from four technical replicates (with repeated sampling from one chemostat per strain). αKG, α-ketoglutarate.

10.1128/mSystems.00170-18.4 FIG S3 Anticorrelated expression of isozymes is conserved across diverged strains of Saccharomyces cerevisiae. Expression profiles are from a series of diauxic shift experiments conducted in wine, laboratory, and natural isolate strains of S. cerevisiae (42). As described for Fig. 4, these experiments show induction of one member of the pair (yellow) and repression of the other (blue) across the diauxic shift. Intensity data correspond to fold change; genes are grouped into isozyme pairs. Download FIG S3, EPS file, 0.3 MB. Copyright © 2019 Bradley et al. 2019 Bradley et al. This content is distributed under the terms of the Creative Commons Attribution 4.0 International license.

We next sought to assign function to “minor” metabolic enzymes, using the aconitase isozyme *ACO2* as an example of an isozyme that is selectively expressed when glucose is available, and the pyruvate kinase isozyme *PYK2* of an example of the converse, an isozyme selectively expressed in the absence of glucose. Additionally, both *ACO2* and *PYK2* have isozyme paralogs (*ACO1* and *CDC19*) with profound deletion phenotypes but have subtle (*ACO2*) or no (*PYK2*) recorded deletion phenotypes themselves.

### Aconitase 2 is required for efficient “glycolytic respiration.”

Aconitases are iron-sulfur proteins that catalyze the second step of the TCA cycle, taking citrate to its isomer, isocitrate, via aconitate. This reaction does not require redox or nucleotide cofactors and does not take place at a branch point in metabolism; however, it is required for α-ketoglutarate synthesis and TCA cycle turning.

Yeast has two aconitase isozymes, *ACO1* and *ACO2*, both of which are mitochondrial; deletions of *ACO1* and *ACO2* are synthetically lethal (13). In analyzing the microarray data described above, we noticed that *ACO1* is repressed by glucose and expressed on glucose removal, while *ACO2* has the opposite transcriptional pattern. *ACO1* is the “major” isozyme, and its deletion has been shown to be severely defective on respiratory carbon sources, such as glycerol, ethanol, and lactate (44). Its expression in the absence of glucose is consistent with the activation of TCA turning. Given that yeast prefer to ferment in the presence of glucose, the function of the *ACO2* isozyme was unclear, although a high-throughput competitive fitness screen had led to a previous report that an *aco2Δ* strain had a growth defect in minimal medium with glucose (14).

We began our experimental studies with an *aco2Δ* mutant strain by studying its growth in glucose minimal medium. We observed no growth defect during the exponential phase in glucose minimal medium, indicating that residual expression of *ACO1* is sufficient to support synthesis of α-ketoglutarate and associated amino acid products (e.g., glutamate, glutamine, and lysine). Growth of the *aco2Δ* deletion strain, however, saturated earlier than growth of the wild type (wt) in glucose minimal medium (Fig. 4B, inset). This suggests that *ACO2* plays an increasingly important role as glucose becomes limiting.

On limiting glucose, wild-type S. cerevisiae continues to perform glycolysis but, instead of fermenting the resulting pyruvate to ethanol, activates respiration to make more ATP. We refer to this state as “glycolytic respiration,” as distinguished from “gluconeogenic respiration,” in which cells respire using 2- and 3-carbon substrates such as ethanol, glycerol, or acetate (Fig. 4A). We hypothesized that the function of the *ACO2* isozyme is to support glycolytic respiration. To test this hypothesis, we grew the *aco2*Δ strain on minimal medium with trehalose as the carbon source. Trehalose, a glucose-glucose disaccharide, is cleaved extracellularly by S. cerevisiae; this produces glucose at a low rate (45), inducing sustained glycolytic respiration. On trehalose, the *aco2*Δ deletion had a fitness disadvantage of 25% (Fig. 4B), confirming that this aconitase isozyme supports glycolytic respiration. Furthermore, we observed no defect of the *aco2*Δ deletion under conditions of growth on minimal media with gluconeogenic carbon sources (Fig. S4A and B), indicating that the metabolic role of the *aco2*Δ mutant is specific to glycolytic and not gluconeogenic respiration.

10.1128/mSystems.00170-18.5 FIG S4 (A and B) The aconitase 2 deletion mutant *aco2*Δ had no growth defect on minimal media with (A) ethanol or (B) ethanol and glycerol as the carbon source. Data represent comparisons of the levels of growth on trehalose and glucose (Fig. 4B). Error bars represent 95% confidence intervals (*n* = 2 for the wild type; *n* = 4 for mutant *aco2*Δ). (C) Growth of wild-type (wt), *pyk2*Δ, *pyk2*Δ::*CDC19*, and *pyk2*Δ::*CDC19E392A* rescue strains on dihydroxyacetone (*n* = 1) revealed full rescue by *CDC19E392A*, a mutant of *CDC19* that is FBP insensitive, but only incomplete rescue by wild-type *CDC19*. Growth (*y* axis) is expressed as a ratio of the OD_600_ at a given time point to the OD_600_ at time 0. Download FIG S4, EPS file, 0.2 MB. Copyright © 2019 Bradley et al. 2019 Bradley et al. This content is distributed under the terms of the Creative Commons Attribution 4.0 International license.

We also profiled the metabolome of the *aco2*Δ mutant and compared it with that of the wild type, using chemostat culture to maintain steady-state growth on limiting glucose. Consistent with lowered aconitase activity, the aconitate levels were somewhat elevated and α-ketoglutarate depleted (Fig. 4C). We also observed increases in the levels of compounds in the *de novo* NAD^+^ biosynthesis pathway from tryptophan, such as kynurenic acid (Fig. S5). This connection to NAD^+^ biosynthesis aligns with previous observations that a deletion of *bna1* (a key NAD^+^ biosynthetic gene) is synthetically sick with the *aco2*Δ mutant (i.e., the double deletion is less fit than expected from single deletion phenotypes) (46). Further work is required to identify the molecular mechanism underlying this phenotype.

10.1128/mSystems.00170-18.6 FIG S5 Intracellular and extracellular metabolic phenotypes of *aco2*Δ and *pyk2*Δ knockouts. Columns are repeated samples from single chemostats. Ion counts for intra- and extracellular metabolites are provided in Data Set S1 and Data Set S2, respectively. (A) Intracellular metabolomic profiles of glucose- or DHA-grown chemostat cultures of wild-type (wt), *aco2*Δ, and *pyk2*Δ strains. The *pyk2*Δ strains showed a decrease in the levels of glycolytic intermediates and phosphorylated compounds in general but especially of pyruvate and acetyl-CoA. A drop in the ATP level relative to the wild-type level was also observed (upper blue highlight). *aco2*Δ mutants showed increases in the abundance of tryptophan breakdown products (upper red highlight) and a drop in the abundance of nicotinate (lower red highlight), indicating a shift from import of nicotinate to *de novo* biosynthesis of NAD^+^. (B) Extracellular compounds from the same chemostat cultures. The first and fourth groups of columns indicate prerun chemostat media. Wild-type cultures showed substantial excretion of TCA cycle intermediates, glutamine, and adenosine and strong uptake of the vitamins nicotinate and thiamine. The *aco2*Δ mutant showed less uptake of extracellular nicotinate (a precursor to NAD^+^) and greater utilization of thiamine (used chiefly to make acetyl-CoA from pyruvate) than the wild-type strain, while the *pyk2*Δ mutant showed very little uptake of either. The *pyk2*Δ mutant also showed sharply reduced excretion of TCA cycle intermediates and excretion of adenine in place of adenosine, possibly indicating a limitation for five-carbon sugars. Download FIG S5, EPS file, 1.5 MB. Copyright © 2019 Bradley et al. 2019 Bradley et al. This content is distributed under the terms of the Creative Commons Attribution 4.0 International license.

10.1128/mSystems.00170-18.9 DATA SET S1 Ion counts, run order, and scale factors for intracellular metabolites sampled from chemostats. Download Data Set S1, CSV file, 0.02 MB. Copyright © 2019 Bradley et al. 2019 Bradley et al. This content is distributed under the terms of the Creative Commons Attribution 4.0 International license.

10.1128/mSystems.00170-18.10 DATA SET S2 Ion counts, run order, and scale factors for extracellular metabolites sampled from chemostats. Download Data Set S2, CSV file, 0.01 MB. Copyright © 2019 Bradley et al. 2019 Bradley et al. This content is distributed under the terms of the Creative Commons Attribution 4.0 International license.

### Pyruvate kinase 2 is required for efficient growth on three-carbon substrates.

**FIG 5:**
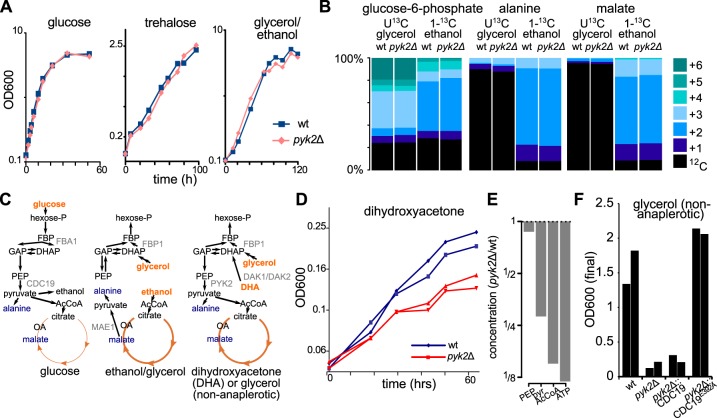
Deletion of the minor pyruvate kinase isozyme *PYK2* results in a selective growth defect on dihydroxyacetone, a three-carbon sugar. (A) Growth of wild-type and *pyk2Δ* strains on glucose, trehalose, and glycerol/ethanol minimal media, revealing no defect for *PYK2* deletion. (B) ^13^C labeling shows that, for wild-type yeast growing on glycerol/ethanol minimal medium, glucose-6-phosphate was labeled from both glycerol and ethanol, but alanine (a proxy for pyruvate) and malate were labeled exclusively from ethanol. Thus, glycerol is not used to make pyruvate. The *PYK2* deletion did not affect these labeling patterns. Labeling patterns represent averages of results from biological triplicates. (C) Schematic of glycolysis and TCA cycle, comparing the metabolism of glucose, glycerol/ethanol medium, and dihydroxyacetone. (D) Growth of wild-type and *pyk2Δ* strains on dihydroxyacetone minimal medium revealed a growth defect for *pyk2Δ*. Data represent results from biological duplicates. (E) At the steady state under conditions of limiting dihydroxyacetone, phosphoenolpyruvate changed only slightly in concentration (87% of wild-type level), while the concentrations of pyruvate (pyr; down 3.5-fold), acetyl-CoA (AcCoA; down 6.7-fold), and ATP (down 8.4-fold) were substantially decreased. Bar plots represent averages of results from four technical replicates (with repeated sampling from one chemostat per strain). (F) Growth on synthetic glycerol (glycerol/CSM-Arg-Asp) medium was normal in the wild-type strain and in the strain in which *pyk2Δ* was rescued with the FBP-insensitive mutant *CDC19^E392A^* but was nearly abolished for *pyk2Δ* and *pyk2Δ* rescued with wild-type *CDC19*. Bars represent the final OD_600_ of biological duplicates.

We next studied growth of the pyruvate kinase isozyme encoded by the *PYK2* gene, an example of an isozyme that is selectively expressed in the absence of glucose. Pyruvate kinase catalyzes the last step of glycolysis, taking phosphoenolpyruvate (PEP) to pyruvate and producing ATP from ADP. This step of glycolysis is highly regulated from yeast (47) to humans (48). The “major” yeast isozyme is known as *CDC19* (where “CDC” is from “cell division cycle”: a *cdc19* deletion causes arrest at the G_1_/S transition). It is expressed in the presence of glucose, and its activity requires high cytosolic fructose-1,6-bisphosphate (FBP) levels, which are produced when glucose is abundant. The *PYK2* isozyme lacks such regulation by fructose-1,6-bisphosphate. Deletion of *CDC19* is lethal on glucose, but deletion of *PYK2* has no known phenotype on either glucose or ethanol (49). Because it does not require activation by fructose-1,6-bisphosphate (FBP), it has been suggested that *PYK2* may contribute to fitness specifically when glucose is limiting (50); however, we found that the *pyk2*Δ deletion exhibits no growth defect on trehalose, arguing against this hypothesis (Fig. 5A). While ethanol-fed cells must also make pyruvate, they appear to do so primarily from the TCA cycle via malic enzyme (*MAE1*) (49), rendering pyruvate kinase unimportant.

S. cerevisiae grows better on a mixture of glycerol and ethanol than on ethanol alone. While this glycerol could potentially be used to make pyruvate via PEP, the *pyk2*Δ deletion strain displayed no growth phenotype on glycerol/ethanol (Fig. 5A). This raised the possibility that in cells fed glycerol-ethanol, as with cells fed ethanol alone, pyruvate is made via *MAE1*. We confirmed this via experiments with [^13^C]glycerol and liquid chromatography-mass spectrometry (LC-MS); upon uniformly feeding the cells ^13^C-labeled glycerol and unlabeled ethanol, while glucose-6-phosphate labeled from glycerol as expected, alanine (which is made by transamination of pyruvate) remained primarily unlabeled (Fig. 5B). In contrast, feeding the cells [1-^13^C]ethanol led to labeling in both malate and alanine. This indicates a lack of reliance on pyruvate kinase in glycerol-ethanol-fed cells. Indeed, the *pyk2*Δ deletion strain showed no difference from the wild-type strain with respect to labeling patterns (Fig. 5B).

In light of the results described above, we hypothesized that *PYK2* would be required on carbon sources that (i) result in insufficient fructose-1,6-bisphosphate levels to activate *CDC19* and (ii) require pyruvate kinase activity to make pyruvate. We further reasoned that 3-carbon substrates would meet those requirements. While glycerol is a common 3-carbon substrate, Saccharomyces cerevisiae cannot grow on glycerol minimal medium without amino acids, presumably because of an inability to maintain cytosolic redox balance (glycerol is more reduced than glucose) (51). We therefore searched for another 3-carbon substrate that could sustain growth as the sole carbon source. S. cerevisiae has two dihydroxyacetone kinases, *DAK1* and *DAK2*, that enable slow but sustained growth on the triose dihydroxyacetone (DHA) (52). DHA enters metabolism through glycolysis/gluconeogenesis, rather than through the TCA cycle, so during growth on DHA, pyruvate should be made using pyruvate kinase rather than malic enzyme. Since *CDC19* is turned off in the absence of glucose (both transcriptionally and allosterically), the majority of the flux through pyruvate kinase should be catalyzed by *PYK2* (Fig. 5C).

Indeed, we observed that deletion of *pyk2* inhibited growth on DHA (Fig. 5D). Further, during continuous culture on dihydroxyacetone, while phosphoenolpyruvate (PEP) levels remained close to wild-type levels, the *pyk2*Δ deletion strain was depleted in pyruvate kinase’s products (pyruvate and ATP). The downstream pyruvate product, acetyl-coenzyme A (acetyl-CoA), was also depleted. This pattern of metabolite levels is consistent with impaired pyruvate kinase activity in this mutant (Fig. 5E; see also Fig. S5).

Further, while laboratory yeast do not grow on a strict minimal medium with glycerol, amino acid supplementation can restore growth. For example, “complete supplement mixture” (CSM), containing selected amino acids and nucleobases, is sufficient to permit growth on glycerol (51). In Saccharomyces cerevisiae, amino acid degradation does not always yield carbon skeletons that can enter central carbon metabolism; instead, the carbon skeletons of many amino acids either are used only in amino acid biosynthesis or are discarded in the form of fusel alcohols via the Ehrlich pathway, which has been speculated to play an important role in maintaining redox balance (53). The only amino acids in CSM known to be catabolized to central carbon intermediates are l-aspartate and l-arginine. To allow growth on glycerol without the confounding influence of other potential carbon sources, we therefore constructed a synthetic glycerol medium supplemented with a version of CSM lacking these amino acids (CSM-Arg-Asp).

During its growth on glycerol with CSM-Arg-Asp, we observed that the *pyk2*Δ deletion strain was severely impaired relative to the wild-type strain (Fig. 5F). In contrast, previous studies have found no such defect in *pyk2*Δ mutants during growth on synthetic complete media containing all amino acids. Together, these results indicate a previously unappreciated role for the pyruvate kinase isozyme *PYK2* in allowing metabolism of three-carbon substrates.

Finally, we wanted to definitively test our hypothesis about the mechanism by which *PYK2* permits growth on three-carbon substrates, that is, that *PYK2* enables growth on glycerol and dihydroxyacetone because, unlike *CDC19*, it does not require allosteric activation by FBP, which is depleted in the absence of glucose. Since *CDC19* expression is repressed under nonfermentative conditions, however, it might also be possible that the presence of either of the two pyruvate kinases allows growth under 3-carbon conditions and that the relevant difference between *PYK2* and *CDC19* is associated with regulation at the promoter level.

To distinguish between these two possibilities, we performed two rescue experiments in which, using the *delitto perfetto* method (54), we kept the promoter of *PYK2* completely intact but replaced the *PYK2* coding sequence with either wild-type *CDC19* or the E392A point mutant of *CDC19*, which allows *CDC19* activity regardless of the FBP concentration (55, 56). Rescue with wild-type *CDC19* did not improve growth on glycerol with CSM-Arg-Asp; rescue with the E392A allele, in sharp contrast, restored growth completely (Fig. 5F). Similar results, including partial rescue by the wild-type *CDC19* allele and complete rescue by the E392A allele, were also observed for dihydroxyacetone (Fig. S4C). These results support our hypothesis that the *PYK2* gene was retained in Saccharomyces cerevisiae because of an “escape from adaptive conflict” (57–59); the presence of *PYK2* allowed the cell to control *CDC19* activity through allosteric activation, which has previously been shown to be important for adapting to short-term glucose removal (60), while also resolving the incompatibility between this regulation and growth on three-carbon substrates.

## DISCUSSION

Through computational analysis of a large compendium of expression data, we found that a set of colocalized metabolic isozymes is differentially expressed in response to glucose availability and that this differential expression is conserved over evolutionary time. We then experimentally found condition-specific contributions to fitness for two of these isozymes, *ACO2* and *PYK2.* In the case of *ACO2*, the deletion shows only a subtle defect on glucose but a defect of 25% on trehalose (i.e., limiting glucose). In the case of *PYK2*, the deletion shows no defect on glucose, ethanol, or glycerol-ethanol but a defect of 42% on dihydroxyacetone and nearly complete growth inhibition when glycerol was the sole carbohydrate source. Also, another study confirmed that the acetyl-CoA synthase isozymes *ACS1* and *ACS2*, identified as part of the same cluster of differentially expressed isozymes returned using our method, display opposite phenotypes on fermentable and nonfermentable carbon sources (61), further supporting the validity of this approach.

The conclusion that even so-called “minor” isozymes make important contributions to fitness that cannot be easily buffered is in line with other recent genome-scale analyses. One recent study first predicted fitness costs for gene deletions using a flux balance model, then calculated the evolutionary rates of these genes using sequence analysis, and finally asked whether genes with larger predicted deletion phenotypes evolved more slowly. The authors demonstrated this expected relationship appeared only when isozymes were assumed to be nonredundant, as opposed to being individually dispensable (62). Furthermore, a study analyzing experimentally determined deletion phenotypes concluded that even closely related, nonessential duplicates actually made distinct, condition-specific contributions to fitness, with effect sizes that are likely large enough for purifying selection to have retained them both (63). Finally, another study integrating experimental data with several bioinformatic estimates of functional divergence concluded that hardly any paralogous pairs are truly functionally redundant (64). Together, these lines of inquiry reinforce the conclusion that failure to find fitness differences in standard media does not indicate dispensability; the differences in fitness that led to retention of a gene may often be detected only under very specific growth conditions. As in the examples we provided here, careful analysis of the relevant biochemical pathways may often be required to infer the appropriate environments in which the differences in fitness can be made manifest.

Among the 53 colocalized isozyme pairs that we identified, our analysis was able to associate the differential expression of 37 pairs with an experimental condition. This leaves 16 whose expression levels were strongly positively correlated, suggesting a potential primary role for gene dosage effects. However, few of these 16 pairs catalyze high-flux reactions according to Kuepfer et al. (3), suggesting that even in those cases, dosage may not represent the primary explanation. Furthermore, some metabolic isozymes that did not show gene expression differences in our analysis have been reported to be differentially regulated at the level of protein concentration. For example, *ENO1* and *ENO2*, the cytosolic enolases, display opposite changes in abundance due to glucose availability in assays performed by chromatography followed by activity analysis (65). This is consistent with the observation that deletion of *eno2*, but not *eno1*, caused abnormal cell cycle progression during standard growth (66). However, *ENO1* and *ENO2* were found to be highly correlated across our expression compendium. This may be either because their mRNAs are hard to distinguish by microarray or because their primary regulation occurs at the level of translation, posttranslational modification, or protein stability. Greater availability of RNA sequencing and quantitative proteomics data will therefore be valuable for this type of analysis in the future.

For isozymes that are differentiated by condition-specific expression, dosage may still have played an important role in their initial evolutionary selection. For example, Conant and Wolfe argue that loss of duplicates outside glycolysis may have led to higher flux through glycolysis and thus to a competitive fitness advantage shortly following the whole-genome duplication in yeast (30). In this model, further specialization of isozymes occurred shortly following the whole-genome duplication. Such further specialization likely provided an evolutionary benefit through escape from adaptive conflict; gene duplication allows the expression level and/or enzymatic activity of a protein to be tailored to two different conditions, where a single protein would have to “split the difference” and would thus be imperfectly adapted to both (58). Note also that dosage may be more important as a current selective pressure for other classes of duplicated genes, such as those encoding ribosomal proteins, which are overrepresented among whole-genome duplicates (2, 24).

What kinds of adaptive conflict might drive isozyme differentiation? One conflict is between affinity and speed (*K_m_* versus *k*_cat_), as exemplified by the hexose transporters. Another kind of conflict arises from differing allosteric regulatory requirements, as exemplified by the pyruvate kinases. Allosteric regulation of pyruvate kinase by fructose-1,6-bisphosphate (FBP) is conserved from bacteria (67) to human (68). Recently, it has been recognized that ultrasensitive (i.e., cooperative) activation by FBP enables pyruvate kinase activity to turn “on” and “off” in a switch-like manner in response to glucose availability (60). In bacteria, similar allosteric activation has also been observed for not only pyruvate kinase but also the other main PEP-consuming enzyme, PEP carboxykinase (56). Such switch-like regulation facilitates growth in oscillating glucose environments and prevents futile cycling in gluconeogenic ones. It is problematic, however, for substrates that enter metabolism via lower glycolysis. They must rely on simultaneous “downward” flux through pyruvate kinase on the one hand and on “upward” flux through fructose-bisphosphate aldolase to produce 6-carbon sugars on the other. “Downward” flux requires high levels of FBP to activate pyruvate kinase, while “upward” flux requires low levels of FBP to render net FBP formation by aldolase thermodynamically favorable. Here, we show that the solution involves expression of *PYK2*, whose activity does not depend on high FBP levels.

It is notable that even though the differential allosteric regulation characteristics of *CDC19* and *PYK2* have been known for 20 years, and despite extensive interest in pyruvate kinase isozymes due to the strong association of the mammalian pyruvate kinase M2 isozyme with cancer (69), no functional role for *PYK2* had been previously identified. Indeed, a recent competitive fitness study assaying more than 400 growth conditions revealed a growth phenotype under at least one condition for 97% of yeast genes but did not find any fitness defect for the *pyk2*Δ deletion (16).

Another study (70) expressed both *CDC19* and *PYK2* from nonnative promoters to tune pyruvate kinase function experimentally, concluding that lower pyruvate kinase activity was accompanied by an increase in oxidative metabolism and oxidative stress resistance. This is in line with previous reports indicating that lower growth rates, such as the authors show occur with pyruvate kinase downregulation, induce both respiration and stress-protective machinery in yeast (20, 71) which includes intracellular glutathione, an important antioxidant (72). However, that work did not describe a specific functional role for *PYK2* and did not provide experimental evidence explaining the retention of *PYK2* in wild-type yeast. Here, we demonstrated that the Pyk2p protein product specifically, and not Cdc19p, is important for efficient growth on three-carbon substrates.

Despite decades of research on yeast physiology, dihydroxyacetone was identified as a sole carbon source for S. cerevisiae only in 2003 (52). Further, even though glycerol is a commonly used nonfermentable carbon source in yeast biology, it has almost always been used in rich or extensively supplemented media, which contain other potential carbon sources (51). A full understanding of the role of yeast isozymes in central metabolism will likely go hand in hand with a more complete understanding of potential modes of carbon metabolism. More generally, many functions of yeast metabolic genes and enzymes may become manifest only when growth and viability are studied in a wider variety of environments. Because prolonged propagation on glucose may have led to the loss of certain metabolic capabilities (e.g., xylose catabolism) in laboratory yeast strains (73), study of natural isolates may also be important.

An important dichotomy in our findings is that between the conditions that control isozyme expression and those in which isozymes are functionally important. A similar duality between the genes induced under a particular condition and the genes necessary for growth in that condition has been previously observed (74–76). Here, we found that expression of a large subset of isozymes is controlled by glucose availability and, indeed, experimentally confirmed that two “minor” isozymes play important roles when glucose is minimally available or absent. Yet this broad characterization in terms of gene expression belies the much more complex and specific functional roles for these isozymes. For example, *ACO2* is expressed at the highest levels in the presence of glucose and yet *ACO2* is functionally important only when glucose is scarce: this indicates that its expression under high-glucose conditions actually reflects preparation for future times when glucose is limiting. Similarly, the absence of glucose induces expression of *PYK2*, and yet *PYK2* is useful only in the case of carbon sources that feed into lower glycolysis, bypassing the metabolite (and key allosteric regulator) fructose-1,6-bisphosphate. Thus, a set of isozymes render yeast central carbon metabolism more flexible, allowing a small number of fundamental transcriptional states to produce optimal enzyme activities across a broad range of potential environmental conditions.

## MATERIALS AND METHODS

### Identifying metabolic isozymes of the same compartment.

Our criteria for identifying isozymes relevant to our study were as follows. (i) The proteins had to perform the same reaction. We drew our initial list of protein pairs meeting these criteria from the reconstructed metabolic model of Saccharomyces cerevisiae iLL672 (3). (ii) The proteins had to have the same small molecules as products and reactants, as detailed in the Yeast Pathway Database (25); we therefore excluded, for example, the protein mannosyl-O-transferases *PMT1* to *PMT6*, since these enzymes have different proteins as substrates. (iii) We included only isozymes annotated as preferring the same cofactors (e.g., NADP+ versus NAD^+^) in the Saccharomyces Genome Database (77). (iv) We considered only those isozymes whose products and reactants were in the same subcellular compartment (i.e., excluding transporters). (v) We excluded isozymes that were annotated to different compartments (e.g., mitochondria versus cytosol) (78) (24 pairs). Our final list comprised 53 isozyme pairs and 85 genes in total, some having two or more partners (e.g., the three glyceraldehyde-3-phosphate dehydrogenases *TDH1*, *TDH2*, and *TDH3*).

To test whether the pathways were enriched for isozymes, the pathways were drawn from the Yeast Pathway database (25) and then combined into the categories shown in Fig. S1 in the supplemental material. (In testing “central carbon metabolism,” we included all reactions from “glycolysis, gluconeogenesis, fermentation,” “pentose phosphate pathway,” and “TCA cycle.”) The proportion of reactions catalyzed by isozymes in each category was calculated, and the two-tailed Fisher’s exact test was used to establish significance (Table S1).

### Assessing anticorrelation of metabolic isozymes.

**(i) Processing of gene expression data.** Microarray data from the Gene Expression Omnibus (GEO) (79) and SPELL (27) were downloaded, processed, and divided into single-experiment data sets as described in Text S1 in the supplemental material. We then constructed an *m*×*n* binary matrix of anticorrelation *B* such that the value corresponding to each entry *b_m,n_* was 1 if gene pair *m* was anticorrelated at a significance threshold *q* value of ≤0.1 in data set *n*. The *B* matrix was sorted by columns from most to least anticorrelation (∑_*m*_*b_m,n_*); the top 10 data sets with the most highly anticorrelated pairs are listed in Table S2. To determine whether the isozyme pairs separated naturally into multiple groups, *B* was also clustered using partitioning around medoids (PAM) with *k*=3 clusters, yielding two coherent clusters (Text S1).

### (ii) Comparison of isozymes with other types of proteins.

We compared the levels of differential expression of isozymes with other pairs of genes. Lists of genes in protein complexes came from a high-throughput pulldown/mass spectrometry assay; only “core” complexes (i.e., sets of proteins that copurified most often) were used (80). We then computed the proportion of arrays in which a given gene pair was differentially expressed, *p_m_*=(∑_*n*_*b_m,n_*)/*n*. Here, as described above, the value corresponding to *b_m,n_* was 1 if the value for correlation *r_x,y,d_* was significantly less than 0 at a *q* value of 0.1 (81) and was set to 0 otherwise; row *m* corresponds to gene pair (*x*, *y*), and column *n* corresponds to data set *d*. The distributions of *p_m_* values were compared via the nonparametric two-tailed Kolmogorov-Smirnov test.

### (iii) Testing differential expression within data set clusters.

For the cluster data sets, per-gene standard deviations were computed for every set. Missing values in the resulting *m×n* matrix, with *m* as the number of genes and *n* as the number of data sets, were imputed using KNNimpute with 10 neighbors (82), discarding first genes and then data sets with more than 70% missing values. Standard deviations were logged after adding a constant equal to half the smallest standard deviation in a given data set. Next, the matrix was first subjected to column normalization (i.e., mean-subtracted and divided by standard deviation) and then to row normalization to ensure that genes or data sets with larger dynamic ranges did not dominate the clustering. This is an approach related to that described by Tavazoie et al. (83).

To ensure the robustness of the clustering, consensus *k*-means clustering (35) was then performed for *k* ranging from 2 to 50. Briefly, in this consensus clustering, 125 subsamples of the original matrix were generated, sampling 80% of the rows and 80% of the columns. These subsamples were clustered via *k-*means, and the resulting clusterings were converted into a “consensus matrix” giving the proportion of subsamples in which two data sets clustered together; this consensus matrix was then hierarchically clustered and cut to give *k* groups. For each value of *k*, Akaike information criterion (AIC) was calculated [i.e., RSS(*k*) + 2*Mk*, where RSS is the residual sum of squares with *k* clusters and *M* is the length of each vector] (84). AIC was then minimized, yielding *k*=16 clusters.

To test for specificity of differential expression within data set clusters, for each isozyme pair (*i_1_*,*i_2_*) and cluster of data sets *C*, we stipulated two criteria. First, we required that the average normalized correlation of the pair (*i_1_*,*i_2_*) tended to be negative in data sets *d* within the cluster *C* as follows:

$$\frac{1}{\left|C\right|}\underset{d\in C}{\sum }\text{atanh}\left(r_{i1,i2,d}\right)\left(\sqrt{N_{d}-3}\right)<0$$

Here, *r_i1,i2_* is the Pearson correlation of isozymes *i*1 and *i*2, and *N_d_* is the number of observations in data set *d*. atanh is the hyperbolic arctangent function, used to perform a Fisher z-transform.

Second, we tested whether the normalized correlation of the pair $z_{i1,i2,d}=\text{atanh}\left(r_{i1,i2,d}\right)\left(\sqrt{N_{d}-3}\right)$ tended to be less within the cluster than outside it, i.e., $z_{i1,i2,\left(d\in C\right)}<z_{i1,i2,\left(d\notin C\right)}$, using a one-tailed rank sum test. *P* values for this test were corrected according to the *q* value method of Storey and Tibshirani (81), and a cutoff *q* value of ≤0.1 was applied.

### Strain construction, media, and growth conditions.

**(i) Strains.** Prototrophic *aco2Δ* and *pyk2Δ* deletion strains were provided by David Hess and Amy Caudy from their prototrophic deletion collection (85). The final prototrophic deletion strains had the genotype *MATα yfg*Δ::*KanMX can1Δ*::*STE3pr-SpHIS5 his*3Δ0 *lyp*1Δ, where *yfg* represents either *aco2*, *pyk2*, or *ho* (for the wild type). For the rescue experiments (Fig. 5E and F), either wild-type *CDC19* or *cdc19-*E392A was introduced into the native *PYK2* promoter using the *delitto perfetto* allele replacement method (54). Starting with DBY12000, a *MAT***a** *HAP1*-positive (*HAP1*^+^) and *GAL2^+^* derivative of FY4 (86, 87), *PYK2* was knocked out using the *pCORE* construct, which contains an antibiotic resistance cassette (KanMX) and a counterselectable marker (*URA3*); selection for resistance to Geneticin yielded a *pyk2*Δ::*CORE* strain. *URA3* was then knocked out to allow use of the counterselectable marker. *CDC19* (wt or E392A) was then amplified using primers with overhangs homologous to this construct. The resulting PCR products were then transformed into the *pyk2*Δ::*CORE* knockout, and transformants were selected based on loss of the counterselectable marker (i.e., resistance to 5-fluoroorotic acid [5-FOA]), yielding pyk2Δ::*CDC19* and pyk2Δ::*cdc19-*E392A strains in a *ura3*Δ background. Finally, these strains were mated to a strain with wild-type *URA3*; sporulation and dissection yielded a fully prototrophic strain with the genotype *MAT***a** pyk2Δ::*CDC19*(wt/E392A). Strains were compared to the DBY12000 wild-type parent strain.

**(ii) Medium recipes.** Minimal media for batch cultures were prepared using 6.7 g/liter yeast nitrogen base (YNB; Difco) and an appropriate carbon source. The final concentrations of carbon sources were 20 g/liter for glucose (YNB-glucose), 100 g/liter for trehalose per Jules et al. (45) (YNB-trehalose), 20 g/liter each for glycerol and ethanol (YNB-glycerol/ethanol), and 9 g/liter (100 mM) for dihydroxyacetone per Boles et al. (49) (YNB-DHA). Minimal media for chemostats were prepared according to the glucose-limited chemostat medium (CM-glucose) recipe reported previously by Dunham and Mitchell (88); for DHA-limited chemostats (CM-DHA), 8 mM DHA was substituted for 8 mM glucose. Synthetic glycerol medium (glycerol/CSM-Arg-Asp.) was prepared using YNB without ammonium and 3% glycerol, plus the following nitrogen bases and l-amino acids: 10 mg/liter adenine, 20 mg/liter His, 50 mg/liter Ile, 100 mg/liter Leu, 50 mg/liter Lys, 20 mg/liter Met, 50 mg/liter Phe, 100 mg/liter Thr, 50 mg/liter Trp, 50 mg/liter Tyr, 20 mg/liter uracil, and 140 mg/liter Val.

**(iii) Batch cultures.** Wild-type (*ho*Δ) and isozyme deletion (*aco*2Δ and *pyk*2Δ) strains were struck out on yeast extract-peptone-dextrose (YPD). For growth curve analyses, a different colony was picked for each biological replication, placed into YNB-glucose, and grown overnight. Overnight cultures were then set back in YNB-glucose for at least one doubling period. For growth curve analyses on glucose, the cultures were then set back such that the optical density at 600 nm (OD_600_) was close to 0.1. For growth curve analyses on YNB-trehalose and YNB-dihydroxyacetone, log-phase cultures grown in glucose were set back into media containing either trehalose or dihydroxyacetone and allowed to double at least twice in the new medium; cultures were then set back to an OD_600_ of 0.1 to start the growth curve analysis. For the experiment measuring final density on glycerol/CSM-Arg-Asp., cultures were inoculated into synthetic dropout (SD) media, washed 3× in YNB without ammonium, and then set back to an initial OD_600_ of 0.05 in glycerol/CSM-Arg-Asp.; the final OD_600_ was measured after 16 days.

**(iv) Chemostat cultures.** Wild-type and isozyme deletions were struck out onto a YPD plate and then inoculated in CM-glucose (for glucose-limited chemostats) or in minimal medium with YNB, dihydroxyacetone, and 0.05% glucose (CM-DHA-glucose, for DHA-limited chemostats). For CM-DHA-glucose experiments, the cultures grown overnight were allowed to grow an additional day. The cultures grown overnight were then used to inoculate one chemostat per strain/medium combination, with media limited for either glucose or for dihydroxyacetone (see medium recipes). Batch mode proceeded for 1 day for glucose-limited chemostats and 3 days for DHA-limited chemostats. The working volume of each chemostat was 300 ml. After the batch mode step, pumps were turned on such that the dilution rate was approximately 0.018/h. Mean and median cell volume and cell number levels were assayed using the Coulter counter. Each chemostat was sampled four times for each of the metabolites after Coulter counter readings, and the medium pH levels were stabilized.

### Metabolite sampling and normalization.

**(i) Metabolite pool size sampling from chemostats.** Metabolites were sampled according to the procedure described previously by Crutchfield et al. (89). A 5-ml volume of chemostat culture was filtered, and metabolism was immediately quenched using 1.5 ml of −20°C 40:40:20 acetonitrile (ACN)-MeOH-H_2_O. Samples were then concentrated by drying with nitrogen gas and subsequent resuspension in 100% high-pressure liquid chromatography (HPLC) water. These samples were then analyzed via reversed-phase ion-pairing liquid chromatography coupled to a Thermo Fisher Scientific Exactive instrument with high mass accuracy, allowing untargeted analysis (90, 91). Samples were collected in quadruplicate. Medium filtrate samples were analyzed using two triple-quadrupole mass spectrometers, one running in positive mode (Finnigan TSQ Quantum Ultra) that was coupled to hydrophilic interaction liquid chromatography (HILIC) (92) and one in negative mode (TSQ Quantum Discovery Max) that was coupled to reversed-phase ion-pairing liquid chromatography (93) as previously described (72).

Data were normalized by total cell volume as described previously by Boer et al. (72) and then log transformed. In Fig. 4 and 5, each sample from a deletion strain was compared to the corresponding wild-type sample run immediately before. In Fig. S5, the data were similarly normalized for run order (which was not confounded with either strain background or nutrient limitation). Briefly, a linear model represented by *y*=*ax+b+ϵ* was fitted to each metabolite vector *y*, using run order as the regressor *x*. The residuals (ϵ) were then kept and visualized as a heat map, after subtracting the average levels of metabolites in the wild-type strain under conditions of glucose limitation.

### (ii) Metabolite labeling experiments.

Metabolites were labeled by transferring cultures into media with either [U-^13^C]glycerol and [U-^12^C]ethanol or [2-^13^C]EtOH and [U-^12^C]glycerol. Labeled substrates were provided by Cambridge Isotopes. After 8 h of growth in the labeled medium, metabolism was quenched, and extracts were then concentrated 3× and analyzed with LC-MS as described above. Three biological replicates were sampled per strain and condition. Isotope labeling patterns were corrected for natural abundance and impurity of the tracer (∼1% ^12^C) using the least-squares method.

### Data availability.

The source code used to perform the analyses is available from http://www.bitbucket.org/pbradz/isozymes. Steady-state metabolite ion counts are provided in Data Set S1 and Data Set S2 in the supplemental material.
