# Supplementary material for: Minor Isozymes Tailor Yeast Metabolism to Carbon Availability
Source: mSystems. 2019 Feb 26;4(1):e00170-18. doi: 10.1128/mSystems.00170-18 (PMC6392091; doi:10.1128/mSystems.00170-18)
Supplement: TABLE S1 [file mSystems.00170-18-st001.docx]

| **Pathway** | **Isozymes** | **Other** | ***p*-value** |
| --- | --- | --- | --- |
| Amino Acid Biosynthesis | 4 | 78 | 1 |
| **Glycolysis, Gluconeogenesis, Fermentation** | **8** | **12** | **5.6x10^-4^** |
| Lipid and Sterol Biosynthesis | 1 | 21 | 1 |
| NAD+ Biosynthesis | 2 | 16 | 1 |
| Nucleotide Biosynthesis | 2 | 14 | 1 |
| Pentose Phosphate Pathway | 3 | 5 | 0.15 |
| SAM Cycle | 1 | 2 | 1 |
| Storage Carbohydrates | 1 | 8 | 1 |
| TCA and Glyoxylate Cycle | 4 | 8 | 0.092 |
| Other | 25 | 417 |  |
| Total | 51 | 581 |  |
