# Supplementary material for: Minor Isozymes Tailor Yeast Metabolism to Carbon Availability
Source: mSystems. 2019 Feb 26;4(1):e00170-18. doi: 10.1128/mSystems.00170-18 (PMC6392091; doi:10.1128/mSystems.00170-18)
Supplement: TEXT S1 [file mSystems.00170-18-s0001.docx]

Minor isozymes tailor yeast metabolism to carbon availability

Patrick H. Bradley, Patrick A. Gibney, David Botstein, Olga G. Troyanskaya, Joshua D. Rabinowitz.

# Supplemental Text

## Note: Key differences from previous analyses of expression data

Previously used statistics for assessing differential isozyme regulation include compendium-wide correlation (1) and PCoR (partial co-regulation, or the standard deviation of per-experiment correlation statistics) (2). One potential shortcoming of both compendium-wide correlation and PCoR is that they can be biased by the composition of the compendium itself, which, despite its diversity, is itself a highly biased sampling of experimental conditions and transcriptional states (3). If a given isozyme pair were strongly anticorrelated in only a small fraction of conditions assayed in the compendium, both PCoR and especially the overall correlation would tend to be dominated by the remaining conditions, lowering power.

Second, calculating a single overall correlation as in Ihmels et al. (1) also requires gene expression data to be concatenated into a single matrix. This requires normalization to minimize the contribution of technical variation between experiments; however, with hundreds of experiments spanning both single- and dual-channel microarrays, it is unclear how such normalization would be performed. Correlation across an entire compendium has also been shown to compare unfavorably to weighted per-dataset correlations in the context of function prediction (4).

Third, neither PCoR nor the overall correlation provide any condition-specific information, whereas the main purpose of the method we use is to associate the differential expression of isozymes with specific environmental perturbations. Finally, previous methods have not used thresholds derived from statistical hypothesis testing with false-discovery rate correction, which becomes particularly important in a compendium with hundreds of experiments (5), and all of these previous approaches used both positive and negative correlation, making them potentially sensitive to cross-hybridization (6, 7).

In our analysis, we took a different approach in which, within each experiment in our compendium, we identified statistically significant negative correlations between isozymes, and then looked for trends within and across clusters of related experiments.

## Supplemental Methods

### Assembly of microarray compendium and calculation of normalized correlations

The datasets constituting the microarray compendium were drawn from two sources. First, we included 129 datasets previously collected by Hibbs et al. (4). Second, the Gene Expression Omnibus (8) was queried for series uploaded between January 1, 2005 and April 1, 2009 that included array data from *Saccharomyces cerevisiae* and contained at least 6 samples, returning 417 datasets. These 417 were then processed according to the following schema: (i) tabular files were extracted from the SOFT files; (ii) larger datasets were broken into logical datasets as per Hibbs et al. (4); (iii) redundant datasets (e.g., supersets of other logical datasets, or duplicates of the data collected by Hibbs et al.) were excluded; (iv) missing values were imputed via the KNNimpute tool in the Sleipnir library (9, 10) with default parameters; and (v) multiple probes for the same gene were collapsed into per-gene expression profiles using a maximum likelihood method (4). In total, 285 non-redundant expression datasets were added to the compendium from GEO.

Normalized correlations were calculated by taking pairwise Pearson correlations between all pairs of genes *x* and *y* in every dataset *d* as follows:

$$r_{x,y,d}=\sum_{i=1}^{|\left| x \right||} \frac{(x_{i}-\bar{x})(y_{i}-\bar{y})}{\left( \left| \left| x \right| \right|-1 \right)\sigma_{x}\sigma_{y}}$$

Here, *x* and *y* are the gene expression vectors in dataset *d*, *||x||* is the length of x, *x_i_* and *y_i_* are the values of each vector at index (i.e., array) *i*, and *σ_x_* and *σ_y_* are the standard deviations of *x* and *y*. Pearson correlations were then transformed according to the Fisher’s z-transform (i.e., hyperbolic arctangent). These scores were converted to standardized *z*-scores by dividing by the standard error $\left( \sqrt{\left| \left| x \right| \right|-3} \right)^{-1}$. The *z-*scores were then used as the test statistic for a Wald test, yielding *p*-values*.* Finally, the *p-*values were corrected for multiple testing by conversion to *q-*values (5, 11).

### Logistic regression classification

We classified isozyme pairs into two groups based on whether they appeared, based on analysis of expression in our expression compendium, more like members of the same protein complex or more like random pairs. To do this, we took the binary differential expression vectors (see above) for pairs in the same protein complex and for random pairs, and fit a generalized linear model (using the “glm” function in R (12))) to classify pairs as one or the other:

$$\log\left( \frac{c}{1-c} \right)=\beta_{0}+\beta_{1}p_{m}+\epsilon$$

Here, *c* is the probability of a gene pair belonging to the same complex (vs. random pairs), $\beta_{0}$ and $\beta_{1}$ are parameters learned by the model, $p_{m}$ is as above, and $\epsilon$ represents residual error. A value of *c* above 0.5 indicated that the pair was, at least weakly, more likely to be a “same complex” pair, and a value of *c* above 5/6 or below 1/6 was taken as a confident classification of a pair into the “same complex” or “random” categories. A graphical illustration is presented in Supplemental Figure 1.

### PAM clustering

Partitioning around medoids (PAM) was performed using the cluster package in R (13) with *k* = 3 based on a dissimilarity matrix constructed using the Jaccard distance.

### Analysis of evolution asymmetry

Tables of orthologs (“pillars”) for isozymes and their protein sequences were downloaded from Version 7 of the Yeast Genome Order Browser (14). Additionally, *S. pombe* orthologs and their protein sequences were downloaded from Pombase (15) (version date 10/31/18). For each isozyme pair, all orthologs of either gene were aligned together using Clustal Omega, then a tree was constructed using RAxML with automatic amino acid model selection. The trees were rooted using, where possible, the *S. pombe* ortholog as an outgroup; if none was found, the most recent common ancestor (MRCA) of the non-WGD yeasts *L. thermotolerans*, *L. waltii*, and *L. kluyverii* was used, and if this was not possible, the tree was rooted using the MRCA of the next-most-diverged non-WGD yeasts *E. gossypii* and *E. cymbalariae*. An asymmetry score *A* for each *S. cerevisiae* gene pair was then calculated by measuring the root-to-tip distance for both isozymes, then dividing the absolute value of the difference by the mean, i.e.:

$$A_{ij}=\frac{|{RTT}_{i}-{RTT}_{j}|}{\frac{{RTT}_{i}+{RTT}_{j}}{2}}$$

where *RTT_i_* is the root-to-tip distance for one isozyme and *RTT_j_* is the root-to-tip distance for the other. This score is conceptually similar to the “relative nonsynonymous divergence” metric developed by Kim and Yi (16). *A_ij_* measurements for isozyme pairs that were classified as “complex-like” in the logistic regression classification and for those pairs that were classified as “random-like” were compared using a Wilcox rank-sum test.
