# Supplementary material for: Minor Isozymes Tailor Yeast Metabolism to Carbon Availability
Source: mSystems. 2019 Feb 26;4(1):e00170-18. doi: 10.1128/mSystems.00170-18 (PMC6392091; doi:10.1128/mSystems.00170-18)
Supplement: TABLE S2 [file mSystems.00170-18-st002.docx]

| Dataset | Brief description | Anticorrelated isozymes |
| --- | --- | --- |
| Meng (NP) | sua5Δ deletion vs. wildtype | MET12/MET13, HXK1/HXK2, HXK2/GLK1, SAM2/SAM1, PDC6/PDC5, PDC6/PDC1, DLD1/DLD2, TDH1/TDH3, URA5/URA10, UTR1/YEF1, ACS1/ACS2, CDC19/PYK2, DAK2/DAK1, ENO1/ERR3, ENO2/ERR3, GND2/GND1, GPM1/GPM2, GPM2/GPM3, GPP2/GPP1, IMD2/IMD4, PFK26/PFK27, PGM1/PGM2, SOL3/SOL4, TAL1/NQM1 |
| Smith 2008 | Growth of segregants on ethanol and glucose | HXK1/HXK2, HXK2/GLK1, PDC6/PDC5, PDC6/PDC1, PYC1/PYC2, NMA2/NMA1, ACO1/ACO2, YIA6/YEA6, DAL7/MLS1, ENO1/ERR3, ENO2/ERR3, GND2/GND1, GPD1/GPD2, GPM1/GPM2, GPM2/GPM3, INM1/INM2, MHT1/SAM4, PFK26/PFK27, PGM1/PGM2, SOL3/SOL4, TAL1/NQM1, URA7/URA8 |
| Rossouw 2008 | Profiling of wine strains during fermentation | HXK1/HXK2, HXK2/GLK1, PDC6/PDC1, PDC5/PDC1, LYS21/LYS20, URA5/URA10, ACO1/ACO2, ACS1/ACS2, CDC19/PYK2, DAL7/MLS1, ENO1/ENO2, ENO2/ERR3, GDH3/GDH1, GND2/GND1, GPM1/GPM3, GPM2/GPM3, PGM1/PGM2, SOL3/SOL4, TAL1/NQM1 |
| Zhu 2009 | Heat shock | HXK1/HXK2, HXK2/GLK1, PDC6/PDC5, PDC6/PDC1, PYC1/PYC2, URA5/URA10, NMA2/NMA1, ACO1/ACO2, ACC1/HFA1, ACS1/ACS2, ASN1/ASN2, GDH3/GDH1, GPD1/GPD2, GPM1/GPM3, GPM2/GPM3, GPP2/GPP1, PFK26/PFK27, PGM1/PGM2, URA7/URA8 |
| Urban 2007 | Sch9/TORC1 signaling | NTH2/NTH1, PDC6/PDC5, PDC6/PDC1, PYC1/PYC2, ADE16/ADE17, UTR1/YEF1, ACC1/HFA1, ACS1/ACS2, DAK2/DAK1, DAL7/MLS1, ENO1/ERR3, ENO2/ERR3, GDH3/GDH1, GND2/GND1, HMG2/HMG1, PFK26/PFK27, SOL3/SOL4, TAL1/NQM1, URA7/URA8 |
| Guldal (NP) | Ras/PKA response (glucose regulation) | MET12/MET13, HXK1/HXK2, HXK2/GLK1, PYC1/PYC2, URA5/URA10, THI20/THI21, NMA2/NMA1, ACO1/ACO2, YIA6/YEA6, ACS1/ACS2, GDH3/GDH1, GPD1/GPD2, GPM1/GPM3, GPM2/GPM3, INM1/INM2, MHT1/SAM4, PFK26/PFK27, SOL3/SOL4, URA7/URA8 |
| Sill (NP) | Histone acetyltransferase study (ESA1) | PDC6/PDC5, URA5/URA10, NMA2/NMA1, CDC19/PYK2, DAK2/DAK1, ENO1/ERR3, ENO2/ERR3, GDH3/GDH1, GND2/GND1, GPM1/GPM2, GPM2/GPM3, IMD2/IMD3, IMD2/IMD4, INM1/INM2, MHT1/SAM4, PFK26/PFK27, PGM1/PGM2, SOL3/SOL4, TAL1/NQM1 |
| Singh 2005 | Dessication and rehydration on limiting glucose (lab strain, series 2) | MET12/MET13, SAM2/SAM1, PDC6/PDC5, PDC5/PDC1, PYC1/PYC2, URA5/URA10, NMA2/NMA1, ACS1/ACS2, CDC19/PYK2, GDH3/GDH1, GND2/GND1, GPM1/GPM2, HMG2/HMG1, INM1/INM2, PFK26/PFK27, TAL1/NQM1, THI21/THI22, URA7/URA8 |
| Sha 2013 | Oxidative stress | MET12/MET13, PDC6/PDC5, PDC6/PDC1, URA5/URA10, UTR1/YEF1, NMA2/NMA1, YIA6/YEA6, ACS1/ACS2, ASN1/ASN2, ENO1/ERR3, ENO2/ERR3, GND2/GND1, GPD1/GPD2, GPP2/GPP1, INM1/INM2, MHT1/SAM4, TAL1/NQM1, URA7/URA8 |
| Capaldi 2008 | *HOG1* mutants in either KCl or glucose upshift | HXK1/HXK2, HXK2/GLK1, PDC6/PDC5, PYC1/PYC2, URA5/URA10, ACO1/ACO2, ACS1/ACS2, GDH3/GDH1, GND2/GND1, GPD1/GPD2, GPM1/GPM3, HMG2/HMG1, INM1/INM2, PFK26/PFK27, PGM1/PGM2, SOL3/SOL4, URA7/URA8 |
